# Development, integration and use of an ultra-high-strength gradient system on a human-size 3 T magnet for small animal MRI

**DOI:** 10.1371/journal.pone.0217916

**Published:** 2019-06-03

**Authors:** Kuan-Hung Cho, Sheng-Min Huang, Chang-Hoon Choi, Ming-Jye Chen, Hsuan-Han Chiang, Richard P. Buschbeck, Ezequiel Farrher, N. Jon Shah, Ruslan Garipov, Ching-Ping Chang, Hsu Chang, Li-Wei Kuo

**Affiliations:** 1 Institute of Biomedical Engineering and Nanomedicine, National Health Research Institutes, Miaoli, Taiwan; 2 Department of Biomedical Engineering and Environmental Sciences, National Tsing Hua University, Hsinchu, Taiwan; 3 Institute of Neuroscience and Medicine 4, INM-4, Forschungszentrum Jülich, Jülich, Germany; 4 Institute of Neuroscience and Medicine 11, INM-11, Forschungszentrum Jülich, Jülich, Germany; 5 JARA–BRAIN–Translational Medicine, Aachen, Germany; 6 Department of Neurology, RWTH Aachen University, Aachen, Germany; 7 MR Solutions Ltd., Guildford, United Kingdom; 8 Department of Medical Research, Chi Mei Medical Center, Tainan, Taiwan; 9 Institute of Medical Device and Imaging, National Taiwan University College of Medicine, Taipei, Taiwan; Henry Ford Health System, UNITED STATES

## Abstract

This study aims to integrate an ultra-high-strength gradient coil system on a clinical 3 T magnet and demonstrate its preclinical imaging capabilities. Dedicated phantoms were used to qualitatively and quantitatively assess the performance of the gradient system. Advanced MR imaging sequences, including diffusion tensor imaging (DTI) and quantitative susceptibility mapping (QSM), were implemented and executed on an *ex vivo* specimen as well as *in vivo* rats. The DTI and QSM results on the phantom agreed well with those in the literature. Furthermore, studies on *ex vivo* specimens have demonstrated the applicability of DTI and QSM on our system to probe microstructural changes in a mild traumatic brain injury rat model. The feasibility of *in vivo* rat DTI was also demonstrated. We showed that the inserted ultra-high-strength gradient coil was successfully integrated on a clinically used magnet. After careful tuning and calibration, we verified the accuracy and quantitative preclinical imaging capability of the integrated system in phantom and *in vivo* rat brain experiments. This study can be essential to establish dedicated animal MRI platform on clinical MRI scanners and facilitate translational studies at clinical settings.

## Introduction

Magnetic resonance imaging (MRI) is a powerful, non-invasive medical imaging modality for both clinical diagnosis and preclinical research. In preclinical studies, higher magnetic field strengths, such as 7 T, 9.4 T or higher, have been widely used to achieve superior spatial resolution and signal-to-noise ratio (SNR), providing improved image quality and/or shortening the overall acquisition time. Although studying animal models at ultra-high field is beneficial from the viewpoint of elucidating the underlying mechanisms of diseases, it is also important to transfer information from the animal results to clinical practice, or *vice versa*. Therefore, a cross-platform allowing flexible and efficient translational study is required.

A number of strategies to conduct small animal imaging on clinical MRI systems by adopting either the original coils or inserted gradient coils with customized radio-frequency (RF) coils have been proposed in the literature. For example, Guzman *et al*. studied the feasibility of *in vivo* rat brain imaging on a 1.5 T clinical system with clinically used RF coils [1]. In a subsequent study, a customized RF coil was built and used to acquire structural images of rat brains on a 3 T clinical scanner [2]. More interestingly, diffusion-weighted imaging (DWI) of *in vivo* rat brain was realized using a clinical gradient system on a 1.5 T scanner [3], and fiber tracking of *in vivo* rat brain DWI data has been achieved by inserting a high-strength gradient on a clinical 3 T scanner [4]. Furthermore, quantitative proton spectroscopy has been reported on a clinical 3 T MRI system [5]. Recently, a mouse model of neuroblastoma was investigated using a dedicated RF coil installed in a 3 T clinical system [6]. All the aforementioned studies tested the feasibility of performing small animal experiments by employing clinical whole-body magnets.

Given the increasing interest in translational research aiming to bridge animal models and clinical interpretation, high-strength gradient coils dedicated for small animal use on clinical MRI systems are potentially useful for enhancing the compatibility of sequence development and improving structural and quantitative imaging quality with regard to animal models. Important technical challenges must be addressed in the system integration with a clinical MRI magnet. First, the mounting and fixation of the gradient coil must be designed carefully to reduce vibration-induced motion artifacts during scans with high gradient strengths [7]. Second, the shimming process is a critical step for improving the static field homogeneity as well as image quality. Third, the RF system, including RF coils and interface, plays a dominant role in providing sufficient SNR and spatiotemporal resolution. With an optimized RF coil design, the filling ratios can be maximized and the signal quality can be improved [8].

Therefore, in this study, we aimed to integrate a dedicated ultra-high-strength gradient insert on a human-size 3 T magnet. The integration and performance of the gradient system, together with purpose-built RF coils, were evaluated qualitatively and quantitatively using dedicated phantoms and *ex vivo* brains. Furthermore, the capability of examining *in vivo* small animal studies was demonstrated with the use of advanced MR imaging sequences, including diffusion tensor imaging (DTI) and quantitative susceptibility mapping (QSM). Since the hardware components utilized for integration in our system are selected independently from those of the major system vendors, this study could be useful for those who want to conduct small animal imaging experiments on clinical whole-body magnets.

## Materials and methods

### System integration and hardware setup

**Fig 1:**
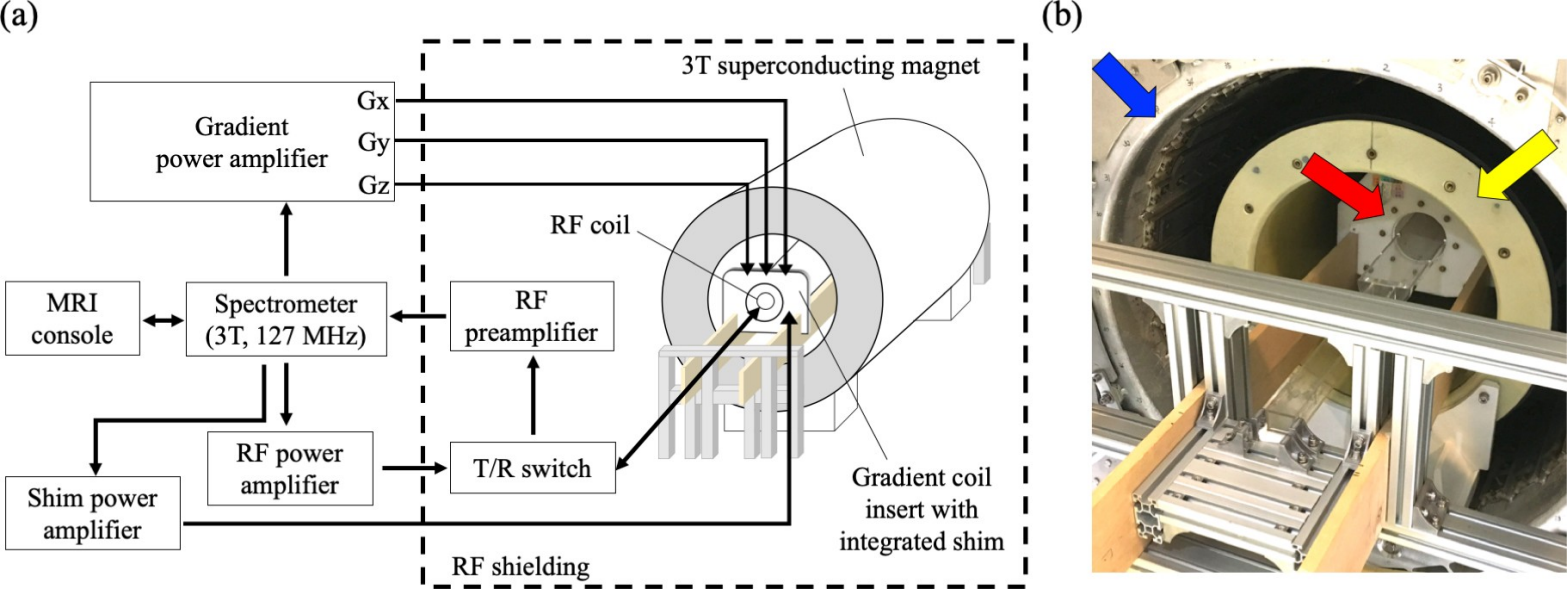
The integrated small animal system. (a) Schematic of overall system configuration, and (b) Integrated high-strength gradient coil (red arrow) for imaging small animals, which is located inside a clinical 3 T magnet (blue arrow) and a human-sized gradient coil (yellow arrow). The mechanical support structures (wood and aluminum) and acrylic animal holder are also shown in the photo.

Fig 1(A) shows an overview of our integrated small animal gradient system. The magnet (Intermagnetics General Corp., Latham, NY, USA) of a used clinical 3 T MRI system (Achieva, Philips Healthcare, Best, Netherlands) was chosen as the base platform. An actively-shielded ultra-high-strength gradient coil set (red arrow in Fig 1(B)) with the maximum strength of 675 mT/m and maximum slew rate of 6750 mT/m/ms (BFG 200/115 S14, Resonance Research Inc., Billerica, MA, USA) was installed and used as the preclinical mode. The dimensions of the small animal gradient coil are 200 mm (outer diameter), 116 mm (inner diameter) and 739 mm (length). Mechanically, the small animal gradient coil was fixed using wooden and aluminum structures onto the 3 T magnet as shown in Fig 1(B). The gradient coils were driven using commercially available gradient power amplifiers (Model 781, Analogic, Peabody, MA, USA). A total of fourteen integrated high-order shim channels of the gradient coil were powered by a shim power supply (MXB-6, Resonance Research Inc., Billerica, MA, USA). All gradient coils and gradient power amplifiers were water-cooled. Both human and animal gradient coils share the power cables and cooling water pipes so the connectors need to be switched between different modes. Temperature control and cooling flow rates were maintained according to the specifications of the gradient power amplifier and the gradient coil.

Note that a human-size gradient coil (outer/inner diameters = 640/480 mm) is also mounted inside the same 3T magnet to be used as the clinical mode, as indicated by a yellow arrow in Fig 1(B). The details about the human gradient system is elsewhere [9]. The human-size gradient coil stays inside the magnet all the time; only the small animal gradient coil set is inserted and removed, while using it for a preclinical mode. Therefore, installing or removing is only required for the small animal gradient insert. Importantly, the small animal gradient coil weighs approximately 35 kg, and the swapping the system mode between human and animal takes less than 15 minutes by two operators.

A linearly polarized, transmit and receive (T/R) litz volume coil with a diameter of 40 mm (Doty Scientific, Columbia, SC, USA) was, for example, used in the experiments for system calibration and phantom validation. A single-loop surface coil with a diameter of 35 mm (Doty Scientific, Columbia, SC, USA) was used in *in vivo* and *ex vivo* scans. The RF chain consisted of a 2-kW RF power amplifier (Communication Power Corp., Hauppauge, NY, USA), a home-built passive-mode T/R switch to direct the RF signal pathways, and two commercial RF preamplifiers connected in series (Advanced Receiver Research, Harwinton, CT, USA). The gradient system, RF system, and all imaging sequences were controlled using a spectrometer operated at the center frequency of 127.7 MHz (EVO, MR Solutions Ltd., Guildford, Surrey, UK). The system software and user interface provided with the spectrometer (PowerScan, MR Solutions Ltd., Guildford, Surrey, UK) was used to control the sequence parameter setting, execution, and image display.

### MR experiments for system calibration and phantom validation

#### Gradient output calibration

**Fig 2:**
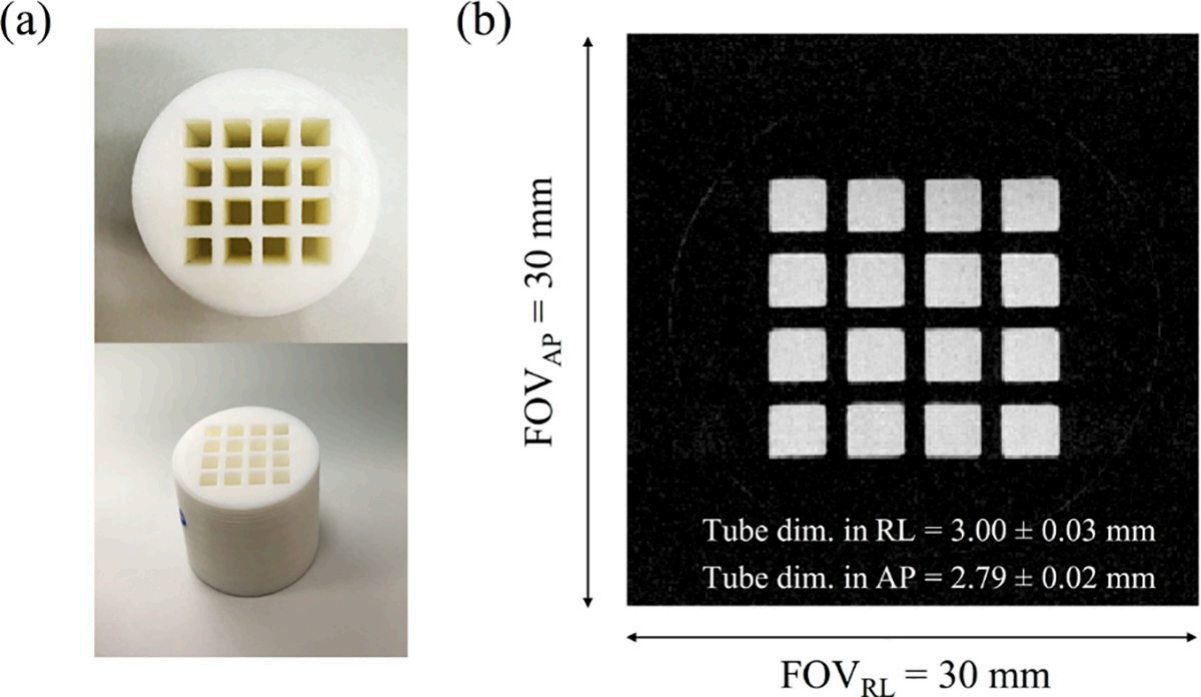
The design of structure phantom for gradient output calibration. A 3D-printed phantom photo including 16 cuboid tubes which was utilized to examine geometrical accuracy. To measure the 3D-printed phantom, the dimension of each tube was set to 3.00 mm along the RL direction and 2.80 mm along the AP direction. Correspondingly, the averaged tube dimension measured from the spin-echo image is 3.00 mm along the RL direction and 2.79 mm along the AP direction.

The gradient output was examined and calibrated using a 3D-printed structure phantom with 16 cuboid tubes, as shown in Fig 2. The dimension of each tube was 3.0 mm × 2.8 mm × 20.0 mm, and all tubes were arranged in a 4 × 4 array. All cuboid tubes were filled with distilled water and sealed for imaging experiments. A spin-echo sequence was executed to acquire anatomical images with the following parameters: field-of-view (FOV) of 30 mm × 30 mm, 3 axial slices with slice thickness of 2 mm, bandwidth of 20 kHz, matrix size of 256 × 256, repetition time (TR) of 2000 ms, echo time (TE) of 22 ms, and number of average (NEX) of 2. Imaging slices were positioned perpendicular to the longest side of the tubes, yielding a total of 16 rectangles on the imaging plane. The dimension of the phantom measured from the image was compared with the actual dimension of the phantom to evaluate the output gradient strength.

#### Slice-profile measurement

The slice profile excited by the applied RF pulse was evaluated using a 50-mL centrifuge phantom filled with copper sulfate solution (copper concentration of 3.08 mmol/L). A five-lobe since excitation pulse was used for the measurement of slice profile. The data was acquired with following sequence parameters: readout FOV of 30 mm, bandwidth of 100 kHz, sampling points of 512, and NEX of 1. In order to investigate the relationship between the applied slice thickness and slice profile, five slice thickness settings (0.5, 1, 2, 3, 4, and 5 mm) were chosen, and correlation analysis was employed.

#### Measurement of T_1_ and T_2_ relaxation times

To examine the accuracy of T_1_ and T_2_ measurements conducted using the integrated system, the CuSO_4_ phantom mentioned above was also scanned using the litz volume coil. In the T_1_ measurement, an inversion recovery spin-echo sequence with various inversion time (TI) was acquired separately with the following parameters: FOV of 40 mm × 40 mm; single slice with slice thickness of 2 mm; matrix size of 64 × 64; NEX of 1; TE of 16 ms; TR of 3000 ms; and TI of 200, 240, 280, 320, 360, 400, 500, 600, 700, 800, 900, 1000, 1200, 1400, 1600, 1800, 2000, and 3000 ms. For the T_2_ measurement, a multi-echo spin-echo sequence was acquired with the same parameters as used for T_1_ measurement, except TE of 25 to 500 ms with a step of 25 ms. In order to obtain the reference values of T_1_ and T_2_ with the same field strength (127.7 MHz), the same solution was prepared in a larger phantom bottle and scanned on another clinical 3 T MRI system (Achieva 3 T, Philips Healthcare, Best, Netherlands). The sequences and imaging parameters except the FOV are the same as used in the experiments for T_1_ and T_2_ relaxation time measurement on the proposed small animal gradient system. The FOV was set to 120 mm × 120 mm owing to the size of phantom. For the calculation of T_1_ relaxation time, the inversion recovery spin-echo signals with various TIs were fit to the equation suggested by Barral et al. [10]:

$$S\left(TI\right)=\left|a+be^{-\frac{TI}{T_{1}}}\right|$$ (1)

For the calculation of T_2_ relaxation time, the first echo was excluded because the second and the later echoes contain a stimulated echo contribution in addition to spin echo, results in an overall increase in the calculation of T_2_ relaxation time [11] and thus signals after the second echo were used for mono-exponential fitting to the following equation for the calculation of T_2_ relaxation time:

$$S\left(TE\right)=ae^{-\frac{TE}{T_{2}}}$$ (2)

#### Measurement of apparent diffusion coefficient

The accuracy of probing water molecular diffusion was evaluated by measuring the apparent diffusion coefficient (ADC) of a distilled-water phantom. DWIs were acquired using a pulsed-gradient spin-echo (PGSE) sequence along three diffusion-encoding directions (readout, phase encoding, and slice). To minimize bias in the ADC measurement due to cross-terms between imaging and diffusion gradients, DWIs with diffusion-encoding directions opposite to the readout, phase encoding, and slice directions were additionally acquired. DWIs with theoretical b-values of 0, 200, 400, 600, 800, and 1000 s/mm^2^ were acquired along each diffusion-encoding direction. Other parameters were FOV of 40 mm × 40 mm, slice thickness of 2 mm, matrix size of 64 × 64, TR/TE of 3000/27 ms, Δ/δ of 15/3.5 ms, and NEX of 2. During the scan, the phantom temperature was monitored using a fiber optical thermometer, and the recorded temperature ranged from 19.2 to 19.3°C. To accurately measure ADC, cross-term-free diffusion signals were first obtained by taking the geometric mean value of the diffusion echo signals with opposite diffusion gradient directions [12]:

$$S_{free}=\sqrt{S_{+d}\times S_{-d}}$$ (3),

where S_free_ is the cross-term-free diffusion signal, and d stands for readout, phase encoding, or slice direction. Although the diffusion cross-term is eliminated by cross-term correction using (3), the constant term due to the imaging gradient cannot be removed. To minimize the error in mapping the ADC value, the constant term from the imaging gradient must be considered in the calculation of the b-value [13, 14], as shown in Table 1. The cross-term-free diffusion signal and the exact b-values were then applied to a mono-exponential decay model for ADC calculation:

$$S=S_{0}\exp\left(-b\times ADC\right)$$ (4),

where S is the diffusion-weighted signal, and S_0_ is the non-diffusion-weighting signal. The ADC calculation was performed using an in-house MATLAB script (The Mathworks, Inc., Natick, MA, USA).

**Table 1:** The b-values used for ADC calculation in readout, phase, and slice directions (unit: s/mm^2^).

b_theory_	b_readout_	b_phase_	b_slice_
0	0.06	0	2.37
200	205.57	205.52	207.89
400	410.89	410.84	413.21
600	617.84	617.79	620.16
800	822.13	822.07	824.45
1000	1028.63	1028.58	1030.95

#### High b-value diffusion MRI measurement

To demonstrate the potential of high gradient strength, we performed diffusion MRI measurements with high b-values on an isopropanol phantom. A PGSE single-shot echo planar imaging (EPI) sequence was used with following parameters: TR/TE of 2000/53 ms, FOV of 30 mm × 30 mm, slice thickness of 2 mm, 10 slices, matrix size of 64 × 64, bandwidth of 200 kHz, NEX of 4, Δ/δ of 25/4 ms, maximum gradient strength of 543 mT/m, b-value of 0 to 8000 s/mm^2^ with a step of 800 s/mm^2^, 1 b0 and 3 diffusion-encoding directions along readout, phase encoding and slice directions, respectively. DWIs with diffusion-encoding directions opposite to the readout, phase encoding, and slice directions were also acquired to minimize cross-terms between imaging and diffusion gradients by using (3).

#### Measurement of phantom susceptibility with QSM

To measure the phantom susceptibility values, an aqueous phantom containing five plastic tubes filled with various concentrations (0.625, 1.25, 1.875, 2.5, and 3.125 mmol/L) of gadolinium chelate (Magnevist, Bayer, Berlin, Germany) was used (Gd phantom). A 3D spoiled gradient-echo sequence with eight echoes of equally distributed TE ranging from 5 to 40 ms was used. The sequence parameters were TR of 60 ms, FOV of 32 mm × 32 mm × 32 mm, matrix size of 128 × 128 × 128, flip angle of 25°, and NEX of 2. To reconstruct QSM, the raw k-space data were processed and used for further analysis.

### MR experiments for *ex vivo* and *in vivo* rat brains

#### Preparation

All animal preparations and related procedures were approved by the Institutional Animal Care and Use Committee at Chi Mei Medical Center (IACUC approved number 105110328). In this study, one *ex vivo* normal rat brain, one *ex vivo* rat brain with mild traumatic brain injury (mTBI) model and three *in vivo* normal rats were scanned to demonstrate the imaging capability of our system. The mTBI rat model was produced by using the fluid percussion approach, as described in a previous study [15]. For *ex vivo* rat brains, rats were deeply anesthetized with urethane and sacrificed by intercardial perfusion with 0.9% phosphate buffer saline solution (PBS), followed by 4% paraformaldehyde/PBS fixative solution. Brains were carefully removed from skulls and postfixed in the same fixative solution for one day at room temperature. For *in vivo* imaging, rats were secured in a customized holder and anesthetized using isoflurane (3% induction and 1.5% maintenance delivered through pure oxygen gas with a constant flow of approximately 600 mL/minute). During the MRI scan, ear and tooth bars were used to fix the rat head to reduce motion-induced image artifacts. A warm water circulation pad was used to maintain the rat body temperature. A pressure sensor (SA Instruments, Inc., NY, USA) was employed to monitor the respiration rate, which was approximately 45 to 55 breaths/minute. After the scan, the rat was placed on a warm electric blanket with a heating lamp to aid recovery from the anesthesia. For the imaging of the *ex vivo* rat brain, a 3D-printed container was filled with agar gel (2%) and the brain was placed inside.

#### DTI with PGSE sequence on *ex vivo* and *in vivo* rat brains

To assess the performance of DTI, we conducted experiments on both *ex vivo* and *in vivo* rat brains using the PGSE DWI sequence. For imaging *ex vivo* rat brains, the sequence parameters were set as follows: TR/TE of 2000/28 ms, FOV of 24 mm × 24 mm, slice thickness of 1 mm, 20 slices, matrix size of 96 × 96, Δ/δ of 15/3.5 ms, NEX of 4, 5 b0 and 60 diffusion-encoding directions equally distributed on a sphere in q-space with the b-value of 1000 s/mm^2^, yielding a total scan time of approximately 14 hours. Dyrby *et al*. demonstrate that diffusion MRI measurements of fixed post-mortem brains were stable over a period up to 1000 days [16]. Since the *ex vivo* rat brains scanned in this study were perfused by pre-mortem intracardial injection and immersed into formalin for only several months before data acquisition, the diffusion properties of these *ex vivo* rat brains would be stable during the experiments. For the imaging of *in vivo* rat brains, the sequence parameters were set as follows: TR/TE of 2000/27 ms, FOV of 40 mm × 40 mm, slice thickness of 1 mm, 20 slices, matrix size of 128 × 128, Δ/δ of 15/3.5 ms, NEX of 1, 5 b0 and 20 diffusion-encoding directions equally distributed on a sphere in q-space with the b-value of 1000 s/mm^2^, yielding a total scan time of 110 minutes. Diffusion tensors were reconstructed from the acquired DWIs by using an in-house program written in C++ [17]. Two DTI quantitative indices, i.e. mean diffusivity (MD) and fractional anisotropy (FA), were calculated. To verify the capability of DTI in our system, DTI-based white matter fiber tractography was reconstructed using a deterministic fiber-tracking algorithm [18]. Two of the callosal areas, including genu (CCg) and splenium (CCs), were tracked and visualized using DSI Studio (http://dsi-studio.labsolver.org). The tracking parameters were: angular threshold of 50°, FA threshold of 0.15, track length shorter than 250 mm, and a total of 3000 tracks to be reconstructed.

#### High b-value DTI with PGSE multi-shot echo planar imaging

To demonstrate the capability of EPI and the advantage of high strength gradient, DTI with high b-value was acquired on an *in vivo* rat brain using the PGSE multi-shot EPI pulse sequence. The sequence parameters were TR/TE of 9000/50 ms, FOV of 25 mm × 25 mm, slice thickness of 1 mm, 20 slices, matrix size of 96 × 96, Δ/δ of 24/3 ms, 2 shots, bandwidth of 200 kHz, 2 b0 and 40 diffusion-encoding directions with b-value of 2000 s/mm^2^, yielding a total scan time of about 13 minutes.

#### Measurement of tissue susceptibility with QSM

A 3D spoiled gradient-echo sequence was employed to acquire raw k-space data for QSM reconstruction for both *ex vivo* and *in vivo* rat brains. For *ex vivo* imaging, 12 echoes spanning the TE range from 5 to 60 ms (5-ms interval) were acquired with a TR of 60 ms, FOV of 28 mm × 28 mm × 16 mm, matrix size of 280 × 280 × 80, flip angle of 25°, bandwidth of 100 kHz, and NEX of 36, yielding a spatial resolution of 100 μm × 100 μm × 200 μm and a total scan time of approximately 15 hours. For imaging *in vivo* rat brains, a total of eight echoes spanning the TE range from 6 to 48 ms (6-ms interval) were acquired with TR of 50 ms, FOV of 30 mm × 30 mm × 20 mm, matrix size of 150 × 150 × 100, flip angle of 30°, bandwidth of 100 kHz, and NEX of 2, resulting in an isotropic spatial resolution of 200 μm and a total scan time of approximately 25 minutes. A first-order flow-compensation module was used to avoid flow-induced artifacts. Note that, since field homogeneity is essential to phase imaging, we chose to perform active shimming to improve the main field homogeneity in this study. To reconstruct tissue susceptibility, we used the morphology-enabled dipole inversion (MEDI) toolbox (http://weill.cornell.edu/mri/pages/qsm.html) for field map estimation, background removal, and field-to-susceptibility inversion [19]. Briefly, the processing pipeline involves the following: first, a field map was obtained by fitting the phase maps derived from multiple echoes, followed by path-based phase unwrapping. Second, the background field was estimated and removed via projection onto the dipole field method, yielding the tissue-related field. Finally, the tissue-related field was used for field-to-susceptibility inversion by using the MEDI algorithm. For a quantitative demonstration, the pixel-wise T_2_* values of the same datasets were also calculated. The susceptibility and T_2_* values of contra-lesional and ipsi-lesional corpus callosum were derived and compared.

## Results

**Fig 3:**
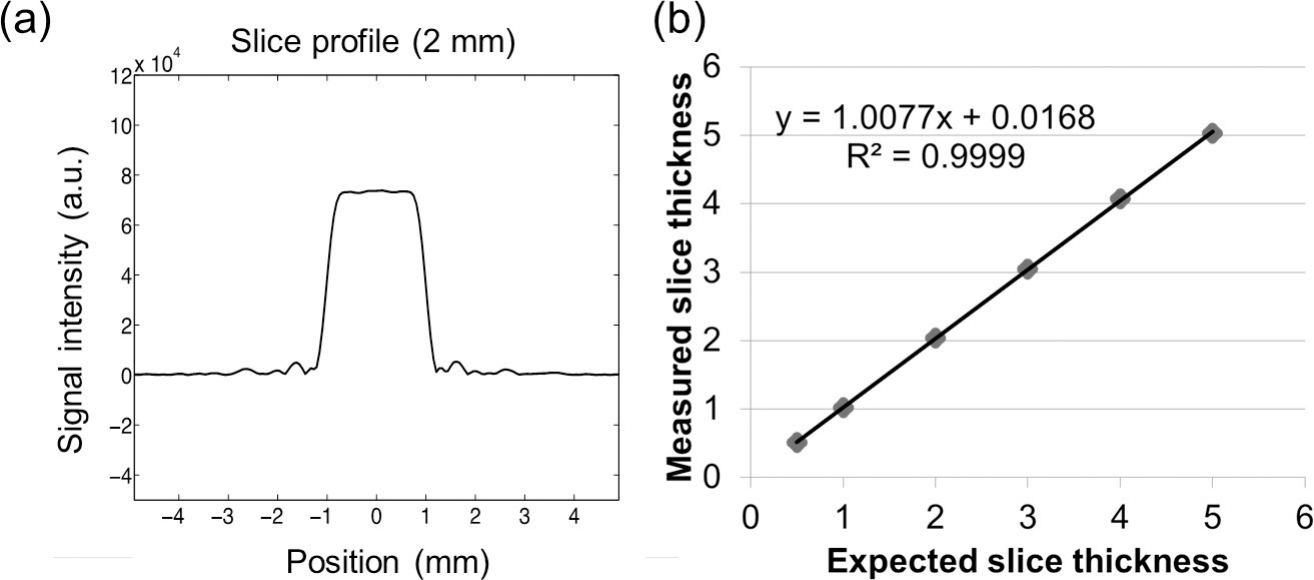
The results of slice profile measurement. (a) Representative slice profile measured using our integrated system corresponding to 2-mm slice thickness. (b) Relationship between expected slice thickness and measured slice thickness, as defined by full-width at half-maximum of the corresponding slice profile. A linear relationship (R^2^ = 0.9999) was found, indicating excellent agreement between the expected and measured values.

Fig 1(B) shows the integrated ultra-high-strength gradient system mounted onto the 3 T magnet, including mechanical support structures and the animal holder. This insertable small animal gradient coil system could increase the feasibility of its use in small animal as well as translational applications. Fig 2 shows the photographs of the 3D-printed phantom with 16 cuboid tubes and geometrical measurement results. In order to evaluate the correctness of slice-selection gradient encoding and RF pulse excitation, the result of a slice-profile measurement (2-mm slice thickness) is displayed in Fig 3(A), which highlights a well-excited slice without severe ringing artifacts. To examine the relationship between the slice thicknesses, we set the sequence and measured the slice thickness from the slice profiles. A scatter plot of the expected slice thicknesses and the full width at half maximum of the slice profiles are shown in Fig 3(B). The linear relationship (R^2^ = 0.9999) indicates excellent agreement between the expected and measured values.

**Fig 4:**
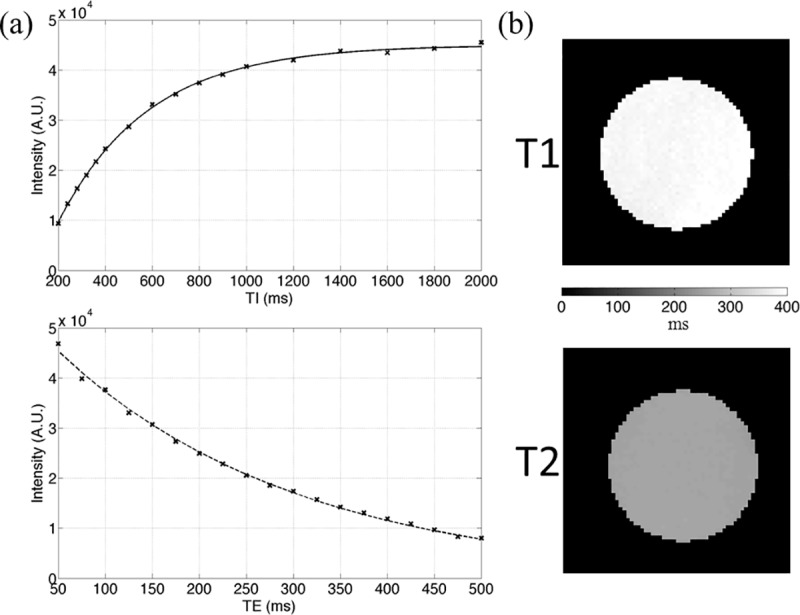
The results of T_1_ and T_2_ relaxation time measurement. (a) Representative curve fitting results of a single pixel. (b) T_1_ and T_2_ maps of CuSO_4_ phantom measured using our integrated system.

Fig 4 presents the quantitative T_1_ and T_2_ maps of the CuSO_4_ phantom. The representative results of single-pixel curve fitting on single-exponential T_1_/T_2_ models are displayed in Fig 4(A). As listed in Table 2, the averaged T_1_ and T_2_ values (± standard deviation) among all pixels within the phantom were 388.6 ± 5.7 and 257.7 ± 1.4 ms, respectively. Compared with the reference T_1_ and T_2_ values of the same CuSO_4_ solution measured using a clinical 3 T MRI system (i.e. 373.5 ± 4.1 and 310.6 ± 1.6 ms).

**Table 2:** Quantitative measurement of T_1_/T_2_ values of CuSO_4_ phantom and ADC values of water phantom.

CuSO_4_ phantom		Water phantom		
T_1_	T_2_	ADC_readout_	ADC_phase_	ADC_slice_
388.6 ± 5.7	257.7 ± 1.4	2.01 ± 0.02	1.97 ± 0.02	1.99 ± 0.02

The unit of T_1_/T_2_ is ms, and the unit of ADC is 10^−3^ mm^2^/s.

**Fig 5:**
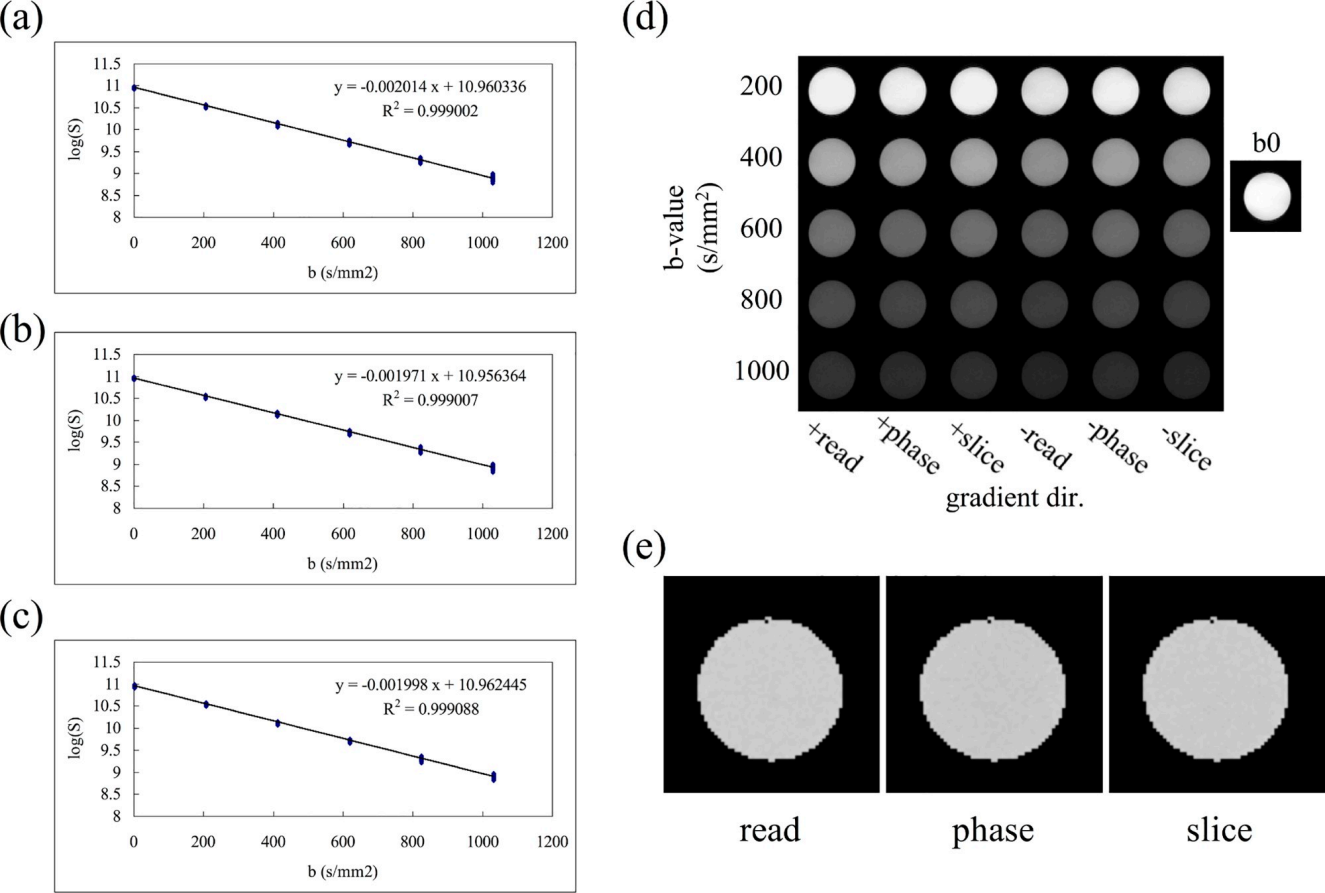
The results of ADC measurement on water phantom. ADC values were estimated by performing linear regression on the plot of logarithm of the cross-term diffusion signals in the region of interest against b-values listed in Table 1. The ADC values are 2.014 *×* 10^−3^, 1.971 *×* 10^−3^, and 1.998 *×* 10^−3^ mm^2^/s along the readout (a), phase encoding (b), and slice (c) directions. The original DWIs (d) showed slightly different signal intensities along different directions owing to the cross-term. The ADC map (e), calculated on a pixel-by-pixel basis by fitting Eq (2), exhibiting favorable uniformity along different directions after cross-term correction. The statistical results from (e) are listed in Table 2.

**Fig 6:**
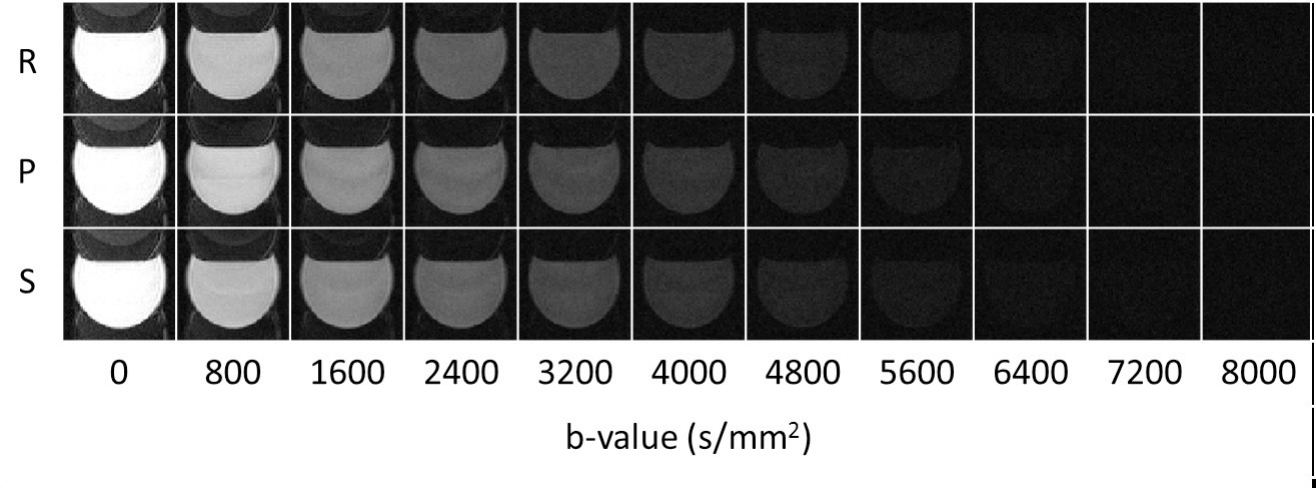
The DWIs with multiple b-values (0–8000 s/mm^2^) of an isopropanol phantom. The diffusion gradients were applied along readout (R), phase encoding (P) and slice (S) directions, respectively. All the image contrasts were adjusted to the same level.

Fig 5 shows the results of ADC measurement conducted using the distilled-water phantom. The logarithms of the cross-term-free diffusion signals within a squared region-of-interest of 400 pixels are plotted against the corrected b-values listed in Table 1. The ADC values along the readout, phase encoding, and slice directions were measured as the slope of the regression line shown in Fig 5(A), 5(B) and 5(C), respectively. As shown in Fig 5(D), the DWIs of multiple b-values along different diffusion-encoding directions exhibit expected signal decays owing to the applied diffusion gradients on this isotropic distilled-water phantom. However, the effect of the cross-terms from the interaction between the imaging and the diffusion gradients can still be observed by comparing the signal intensities along the +slice and–slice directions to those along the remaining directions with the same theoretical b-value. The ADC maps shown in Fig 5(E) were derived on a pixel-by-pixel basis by fitting Eq (2) to the cross-term-free diffusion signals. As listed in Table 2, the ADC values at 19.2°C along the three orthogonal gradient directions are 2.01 ± 0.02, 1.97 ± 0.02, and 1.99 ± 0.02 (10^−3^ mm^2^/s), in good agreement with the theoretical ADC value of 1.98 × 10^−3^ mm^2^/s. Fig 6 shows DWIs with diffusion-encoding gradients (strength of 543 mT/m and b-values of 0–8000 s/mm^2^) along readout, phase encoding and slice directions. The SNR of b0 image is approximately 70.5.

**Fig 7:**
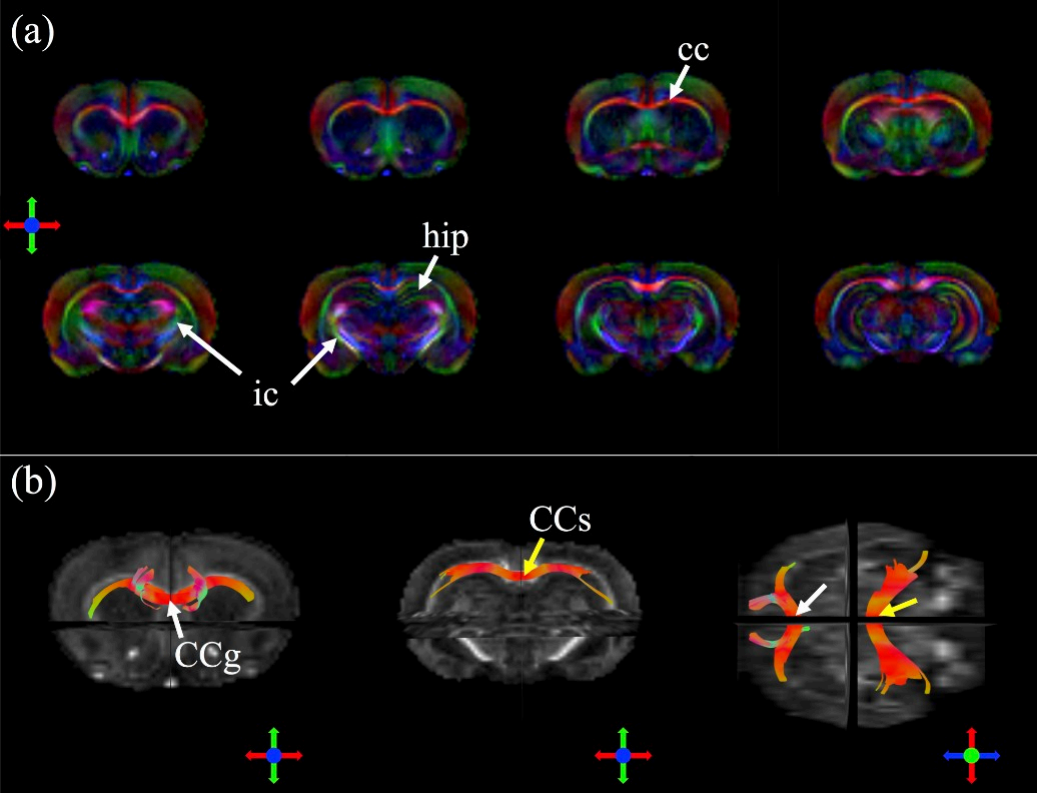
DTI of the *ex vivo* rat brain. (a) High-resolution color-coded FA maps clearly delineate the corpus callosum (cc), internal capsule (ic), and hippocampal formation (hip) clearly. (b) The fiber tractography of the CCg (white arrow) and the CCs (yellow arrow). A horizontal view is shown in the rightmost figure.

**Fig 8:**
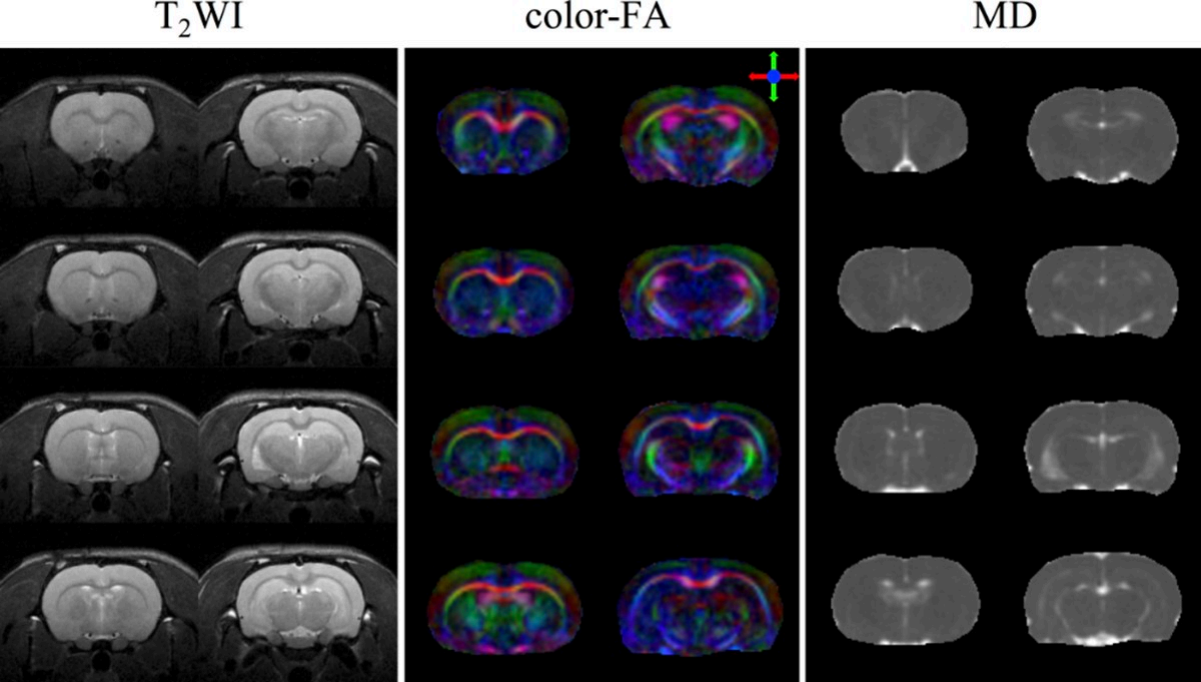
Representative *in vivo* rat brain images. T_2_-weighted image (T_2_WI), fractional anisotropy (FA) map, and mean diffusivity (MD) map are shown. Sufficient SNR and image quality could be obtained using our integrated system, proving its feasibility for conducting *in vivo* experiments on a clinical magnet.

**Fig 9:**
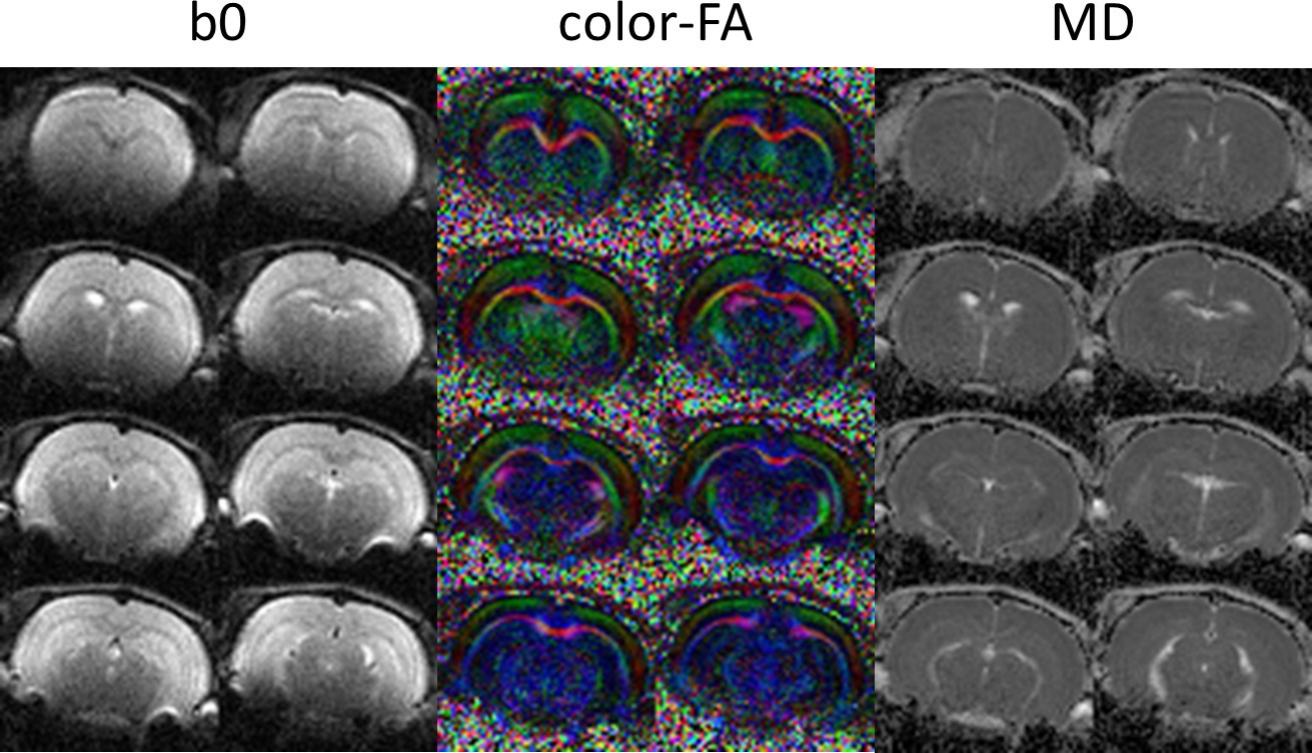
Representative high b-value DTI results of *in vivo* rat brain. Non-diffusion weighting image, color-coded FA map, and MD map are shown to demonstrate the feasibility of EPI acquisition on the integrated system.

Fig 7(A) displays high-resolution color-coded FA maps of an *ex vivo* rat brain. As can be seen clearly, the details of white matter structures, such as the corpus callosum and internal capsule, as well as the hippocampal region, are delineated. The fiber tractography results of the callosum genu (CCg) and the callosum splenium (CCs) overlaid on the FA maps from different viewing angles are shown in Fig 7(B). These suggest that the fiber orientations can be measured accurately from the DWI data acquired using our integrated system. For the in vivo experiment, the T_2_-weighted images and DTI quantitative indices, namely, color-coded FA and MD, of a representative rat are presented in Fig 8, indicating sufficient SNR and image quality were obtained by our integrated system for the *in vivo* experiments. The results of high b-value DTI by using the PGSE multi-shot EPI sequence are shown in Fig 9. The b0 images, color-coded FA and MD of multiple slices are shown to demonstrate the capability of high b-value DTI EPI acquisition on the integrated gradient insert system.

**Fig 10:**
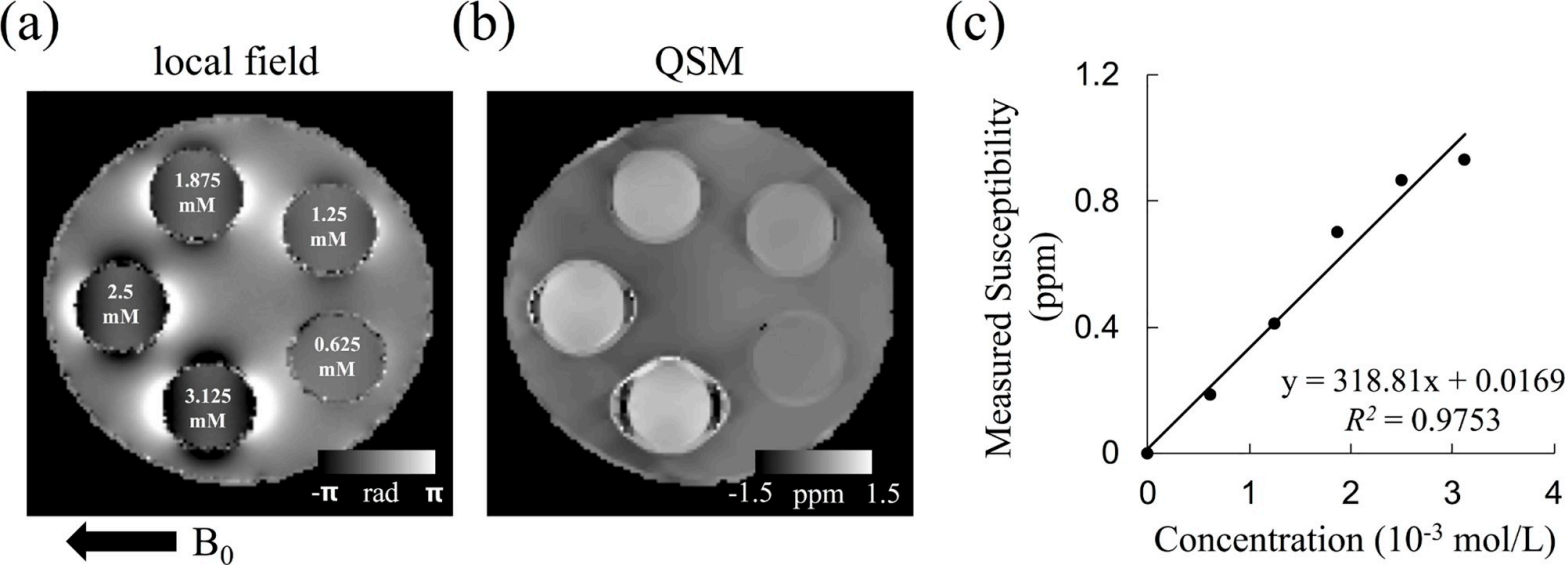
**Maps of local field (a) and QSM (b) of the Gd phantom.** Five different Gd concentrations are included within the phantom. A linear relationship with a concentration-susceptibility slope of 318.81 ppm L/mol was found.

The maps of the local field and the QSM of the Gd phantom are shown in Fig 10(A) and 10(B), respectively. The local field map reveals that the susceptibility dipole effects became stronger as the Gd concentration increased. Moreover, the QSM maps suggest that changes in susceptibility values corresponding to different Gd concentrations could be properly measured using our integrated system. The irregular pattern surrounding the edges of the tubes on the QSM map can be ascribed to the absence of MR signals within the plastic tubes, resulting in imperfections during reconstruction. Although a signal loss occurs in some of the surrounding plastic regions, the linear relationship with the molar susceptibility of 318.81 ppm L/mol (Fig 10(C)) indicates a reasonably precise correspondence between the concentrations of gadolinium and the measured susceptibility values in a homogeneous area within the tubes.

**Fig 11:**
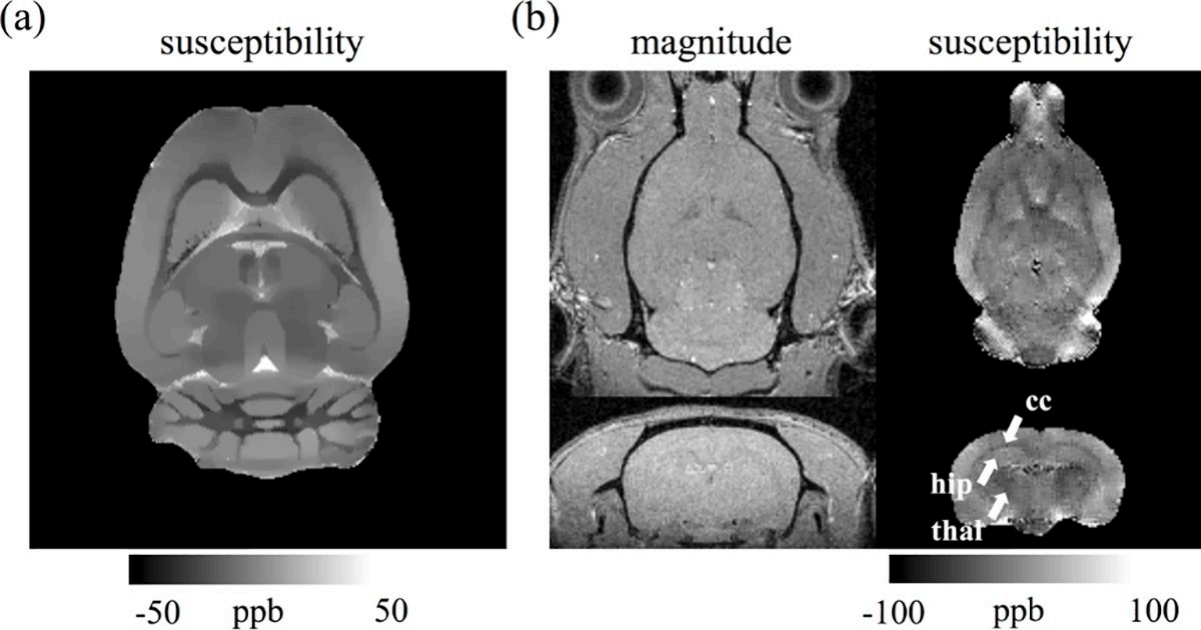
**QSM of (a) *ex vivo* and (b) *in vivo* rat brains.** Our results suggest that the 25-minute scan protocol for the *in vivo* rat brain could reasonably manifest the contrast among corpus callosum (cc), hippocampus (hip), and thalamic area (thal). Note the spatial resolution of *ex vivo* and *in vivo* images was 100 μm × 100 μm × 200 μm and isotropic 100μm, respectively.

To further demonstrate the feasibility of mapping tissue susceptibility, QSM experiments were performed using both *ex vivo* and *in vivo* normal rat brains, as shown in Fig 11(A) and 11(B), respectively. In *ex vivo* QSM, several anatomical structures, such as the corpus callosum, caudate putamen, habenular nucleus, and hippocampus, are clearly delineated in the susceptibility map. In *in vivo* QSM, our results suggest the 25-minute scan protocol could reasonably manifest the contrast among the corpus callosum, hippocampus, and thalamic area.

**Fig 12:**
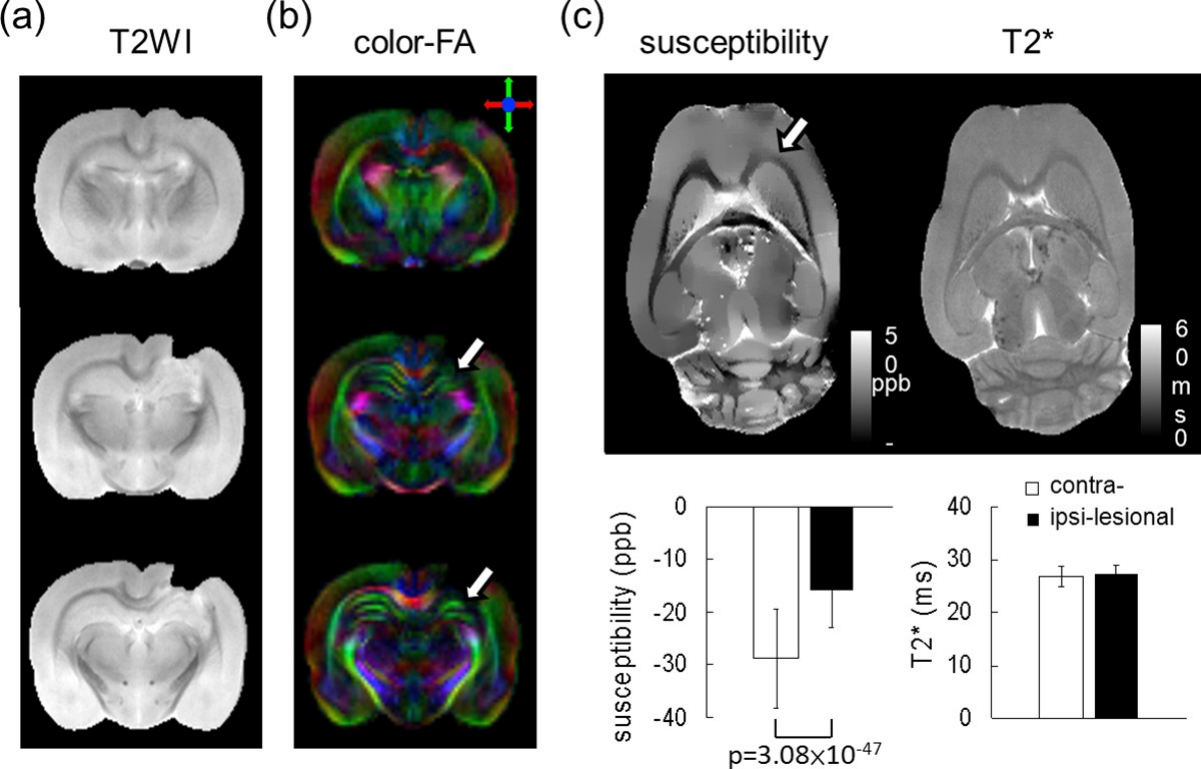
Preliminary examination of the mTBI rat model using our integrated system. (a) T_2_-weighted image (T_2_WI); (b) DTI analysis shows a distinct disruption in hippocampal area, as indicated by the white arrows; and (c) altered white matter integrity of corpus callosum can be seen using QSM, whereas the T_2_* values are insensitive to the changes.

In this study, we additionally investigated the feasibility of our system for preclinical applications pertaining to mTBI rats. The DTI and QSM results of an *ex vivo* mTBI rat brain are shown in Fig 12. From Fig 12(A), the hollowed regions surrounding the lesion site on the T_2_-weighted images indicate the location of concussion in this mTBI model. The concussion-induced edema was cleared during tissue perfusion, resulting in a notch on the *ex vivo* brain. The influence of the concussion on the surrounding white matter structures can be observed in the color-coded FA maps, as displayed in Fig 12(B). A distinct disruption in the hippocampal region of the ipsi-lesional concussion site is indicated clearly by the white arrows. The quantitative susceptibility and T_2_* maps, as well as region-based comparison results, are presented in Fig 12(C). It was found that the susceptibility values in the corpus callosum regions between the contra-lesional and ipsi-lesional sides were significantly different (p = 3.08×10^−47^). Increased susceptibility in the ipsi-lesional corpus callosum area indicates that the affected white matter structures became less diamagnetic, suggesting that disrupted lipid composition or demyelination may play a crucial role in our mTBI model. In contrast, no obvious differences were observed in T_2_* values between the corpus callosum regions on the contra-lesional and ipsi-lesional sides.

## Discussion

In this study, an ultra-high-strength gradient coil insert dedicated for small animal imaging was successfully integrated with a clinical, used 3 T MRI magnet. With systematic system tuning and calibration, the imaging capability of this integrated system was verified qualitatively and quantitatively using a phantom and an *ex vivo* brain sample, as well as by conducting *in vivo* rat brain experiments. A rat mTBI model was used to evaluate the usability of this system by considering both DTI and QSM, which demand high gradient performance and signal phase stability, and the results showed the integrity of altered white matter in the callosal regions. This demonstrates that this ultra-high-strength gradient system has been established successfully and can be potentially useful for future translational research. The advantage of this integrated system is to provide a flexible solution for preclinical research by using dedicated hardware and customized interface. Its imaging capability and the technical challenges associated with integration are discussed in the following sections.

The results of high b-value diffusion MRI measurements on isopropanol phantom demonstrated the advantage of high gradient strength on improving the SNR, consistent with a previous study [20]. However, high gradient strength not only benefits the SNR but also the spatial resolution for imaging the small animals on clinical scanners. For example, achieving the spatial resolution for the high b-value DTI data of *in vivo* rat brain needed the readout gradient strength of 188 mT/m and slice selection gradient strength of 70 mT/m. Such high gradient strengths are not available on most of the clinical gradient systems. Therefore, a strong gradient insert is highly needed for imaging small animals with sub-millimeter spatial resolution on clinical MRI systems.

To verify the accuracy of the quantitative measurement of conventional MRI properties, the T_1_, T_2_, and ADC values of the phantoms were assessed and compared with standard reference values. First, we measured the T_1_ and T_2_ relaxation times of a CuSO_4_ phantom and compared them with the values of the same solution measured using another commercial whole-body clinical 3 T MRI system with the same resonance frequency. Although the same solution was used, the measured T_1_ value using our integrated small animal gradient system was approximately 4% higher than the measured T_1_ value using the commercial clinical system. The coefficient of variation (COV) of the T_1_ values between our integrated small animal gradient system and the commercial clinical system was approximately 2.8%, close to the inter-site variation of T_1_ relaxation time measured on corpus callosum (COV = 2.4%) [21] and smaller than the inter-vendor variability (percent difference of 10.0/7.8/8.6/10.0% for white matter/cortical gray matter/subcortical gray matter/cerebellum) [22].

To examine the feasibility and accuracy of two of the most commonly used physical measures, ADC and susceptibility, MR experiments were performed using distilled water and gadolinium phantoms. The measured mean ADC averaged among the ADC values along three orthogonal axes was approximately 1.99 × 10^−3^ mm^2^/s, which is consistent with the theoretical ADC value at 19.2°C (1.98 × 10^−3^ mm^2^/s). For susceptibility quantification, the slope we calculated from the relationship between Gd concentration and measured susceptibility was 318.81 ppm L/mol, which is consistent with previous findings [23, 24]. Although a few streaking artifacts could be observed on the susceptibility map, they can mainly be ascribed to the phantom design, especially, the wall thickness of the plastic tube, which can be improved through careful design of the susceptibility phantom in future studies. Essentially, our results demonstrated the reliability of QSM measurement using the proposed integrated system. Since the main field homogeneity is a critical factor to have reliable QSM, this result also indicates that the field quality is sustainable with the new gradient insert.

Both the *ex vivo* and *in vivo* DTI experiments on normal rat brains demonstrated the capability of the proposed integrated system in terms of revealing complex tissue fiber structures under different experimental settings. The color-coded FA maps and the fiber tractography of the *ex vivo* rat brain used herein are consistent with the results of a previous study, in which DTI was performed on a 3 T system with an ultra-high-strength gradient coil insert powered by scanner-equipped gradient amplifiers [4]. By comparing *ex vivo* and *in vivo* DTI scans, we found that a higher spatial resolution can be achieved in the *ex vivo* scan for revealing more details pertaining to tissue fiber structures.

Preliminary examinations using DTI and QSM on an *ex vivo* mTBI rat brain were conducted using the proposed integrated system. Hippocampal neurophysiologic changes in the mTBI model have been discussed widely and revealed using DTI, which is a novel imaging modality to probe demyelination and axonal damage [25, 26]. Our results suggest that the DTI on our high-strength gradient system may benefit the mapping of the tissue microstructures under the current experimental settings. A previous report noted that QSM can be used to detect myelin damage of the brain [27]. Moreover, the susceptibility value in the corpus callosum was found to increase after TBI, which might have resulted from a change in lipid fraction (negative susceptibility) in the myelin sheaths. Consistent with the findings in the literature, our QSM analysis shows similar changes in the ipsi-lesional corpus callosum.

The shielding of integrated small animal gradient coil was achieved by a set of shield coils, independent from primary coils and shim coils, to form a self-shielded gradient that produces no significant external field. According to the specification of the gradient coil, the shielding could reduce the eddy current induced residual field to less than 1% after applying a pulse at 50% of maximum current. The DTI results of *ex vivo* rat brains demonstrated reasonable shielding performance since negligible eddy current induced image shift was observed while diffusion gradient was applied with 53% of maximum gradient strength and rise time of 500 μs. Therefore, no further eddy current compensation approach is applied to imaging on our integrated small animal gradient system.

The proposed small animal gradient system can be used on commercial clinical MRI scanners for facilitating translational studies. The control interface and electronics between clinical and preclinical modes can be independent from each other, yielding simpler and faster switching process. Two of the most advanced imaging techniques, DTI and QSM, have been successfully implemented and examined, showing promising ability in small animal research. Although the main concept of this integrated system has been demonstrated, several technical issues remain and will be addressed in future works. First, in order to increase the SNR within an acceptable scan time, an optimized RF coil design (e.g., a phased array coil or a customized-shape coil) should be adapted to meet the needs of different applications. Second, to increase the spatial resolution, the pulse sequences must be well designed and implemented with parallel imaging or simultaneous multiple-slice techniques. Third, the high gradient strength offered by the integrated coil system is potentially beneficial for high-angular-resolution diffusion MRI techniques, which typically require relatively high b-values to resolve complex tissue structures [28]. With higher gradient strengths, the echo times can be further reduced to yield higher SNR for achieving a balance between angular resolution and image quality.

## Conclusions

We successfully integrated an ultra-high-strength gradient system with a clinically used 3 T whole-body magnet. With systematic tuning and calibration, we verified the accuracy and imaging capability of the integrated system in both phantom and rat brain experiments. In addition, we showed that the rat experiments could be conducted successfully under both *ex vivo* and *in vivo* settings. Two advanced MRI techniques, DTI and QSM, were employed on an mTBI rat model, and our preliminary results revealed that trends could be obtained promisingly by comparing the quantitative indices between the contra-lesional and ipsi-lesional sides. Further improvements may be necessary to strengthen the capability of this integrated system and to facilitate its use with a variety of animal applications *in vivo*. This present study could be potentially useful for establishing a dedicated animal imaging platform based on clinical MRI scanners and facilitating translational studies under clinical settings.

## Supporting information

S1 Table The T1 and T2 relaxation times measured on the proposed system and the clinical 3T system. (XLSX)

S2 Table The susceptibility values measured in the corpus callosum regions of the contra-lesional and ipsi-lesional sides. (XLSX)

S3 Table The minimum FOV in readout direction with various bandwidth and gradient strength settings. (DOCX)

S4 Table The minimum slice thickness with various RF pulse duration and gradient strength settings. (DOCX)
