# Supplementary material for: Development, integration and use of an ultra-high-strength gradient system on a human-size 3 T magnet for small animal MRI
Source: PLoS One. 2019 Jun 3;14(6):e0217916. doi: 10.1371/journal.pone.0217916 (PMC6546248; doi:10.1371/journal.pone.0217916)
Supplement: S3 Table — (DOCX) [file pone.0217916.s003.docx]

|  | Minimum FOV in readout direction | | | |
| --- | --- | --- | --- | --- |
| Maximum gradient strength | Bandwidth = 100 kHz | Bandwidth = 125 kHz | Bandwidth = 166 kHz | Bandwidth = 200 kHz |
| 40 mT/m | 5.87 cm | 7.34 cm | 9.75 cm | 11.74 cm |
| 80 mT/m | 2.94 cm | 3.67 cm | 4.87 cm | 5.87 cm |
| 675 mT/m | 0.35 cm | 0.43 cm | 0.58 cm | 0.70 cm |
