# Supplementary material for: Development, integration and use of an ultra-high-strength gradient system on a human-size 3 T magnet for small animal MRI
Source: PLoS One. 2019 Jun 3;14(6):e0217916. doi: 10.1371/journal.pone.0217916 (PMC6546248; doi:10.1371/journal.pone.0217916)
Supplement: S4 Table — (DOCX) [file pone.0217916.s004.docx]

|  | Minimum slice thickness with sinc RF pulse  (time-bandwidth product = 6000 Hz*ms) | | | |
| --- | --- | --- | --- | --- |
| Maximum gradient strength | RF pulse duration = 0.5 ms | RF pulse duration = 1 ms | RF pulse duration = 2 ms | RF pulse duration = 4 ms |
| 40 mT/m | 7.04 mm | 3.52 mm | 1.76 mm | 0.88 mm |
| 80 mT/m | 3.52 mm | 1.76 mm | 0.88 mm | 0.44 mm |
| 675 mT/m | 0.42 mm | 0.21 mm | 0.10 mm | 0.05 mm |
